# To adopt or adapt an existing COS for neonatal research: qualitative study

**DOI:** 10.1186/s13063-026-09834-w

**Published:** 2026-06-08

**Authors:** Jamlick Karumbi, David Gathara, Paula Williamson, Bridget Young

**Affiliations:** 1 Department of Health Data Science, University of Liverpool, Liverpool, UK; 2 Health Systems Research, KEMRI-Wellcome Trust Research Programme, Nairobi, Kenya; 3 Department of Public Health, Policy and Systems, University of Liverpool, Liverpool, UK; 4 Centre for Maternal, Adolescent, Reproductive & Child Health (MARCH), London School of Hygiene and Tropical Medicine, London, UK

**Keywords:** Core outcome sets, Low- and middle-income countries, Adaptation, Kenya

## Abstract

**Background:**

Core outcome sets (COS) standardise the outcomes reported in clinical trials and research, reducing outcome heterogeneity and enabling evidence synthesis. Most neonatal COS have been developed in high-income country (HIC) contexts and may not reflect the priorities, health system capacities, or disease burden of low- and middle-income countries (LMICs). Kenya’s neonatal mortality rate remains high at 21 per 1000 live births, yet no COS exists for neonatal care and research in Kenya or, more broadly, in sub-Saharan Africa. This study aimed to develop a contextually appropriate COS for neonatal care and research in Kenya, and to assess the feasibility of adapting an existing HIC COS for use in an LMIC setting.

**Methods:**

A mixed qualitative and consensus-based approach was used, guided by the COMET handbook. The process comprised three phases: a rapid review of outcomes reported in neonatal trials from sub-Saharan Africa compared with an existing HIC COS; qualitative stakeholder engagement through key informant interviews (KIIs) and focus group discussions (FGDs) with healthcare providers, national-level policymakers, and mothers of previously admitted neonates at two Kenyan hospitals representing urban and rural settings; and an in-person consensus workshop using the nominal group technique with 13 multidisciplinary stakeholders. Thematic analysis followed Braun and Clarke’s six-phase framework. Outcomes endorsed by ≥ 70% of consensus meeting participants were included in the final COS.

**Results:**

Seventeen stakeholders participated in KIIs, and 15 mothers participated in two FGDs. Sixteen candidate outcomes were presented at the consensus meeting. Five outcomes achieved immediate universal consensus: survival, length of hospital stay, ability to feed/weight gain/growth, cognitive ability, and visual impairment/retinopathy of prematurity (RoP). Following discussion and voting, a further seven outcomes were endorsed: impact on mothers and wider family, financial costs to the mother, pain, adverse events due to medicines, respiratory distress, quality of life, and sepsis/infections. The final COS comprises twelve outcomes. Seven overlapped with the existing HIC COS, though with contextually adapted definitions. Five outcomes are Kenya-specific, reflecting the out-of-pocket payment structure, high comorbidity burden, and family-centred care priorities of the Kenyan health system.

**Conclusions:**

Adapting an HIC neonatal COS for use in an LMIC context is feasible, but requires systematic definitional adaptation, engagement with existing local frameworks such as WHO Essential Newborn Care guidelines, and attention to diagnostic capacity constraints. The Kenya COS captures both clinical and life-impact outcomes, reflecting the priorities of diverse stakeholder groups including mothers. Realising its value requires phased implementation sensitive to urban–rural differences in facility capacity, investment in workforce training, and stronger collaboration between clinicians and researchers to ensure outcome measurement serves both care improvement and evidence generation.

**Trial registration:**

This is not a clinical trial. Clinical trial number: not applicable.

**Supplementary Information:**

The online version contains supplementary material available at 10.1186/s13063-026-09834-w.

## Background

Despite global progress in reducing child mortality, neonatal deaths (0–28 days) remain high, particularly in low- and middle-income countries (LMICs). In 2021, 2.3 million neonates died worldwide, with major causes being prematurity, birth complications, and sepsis [1],[2]. Kenya’s neonatal mortality rate (21 per 1000 live births) remains above the Sustainable Development Goal (SDG) target of ≤ 12 per 1000 live births by 2030 [3]. As in most health research, challenges include inconsistent data collection, poor-quality health records, and a lack of standardised outcomes for neonatal care and research [4].

### Why standardised outcomes?

Core outcome sets (COS)—minimum set of standardised outcomes—can improve research comparability, reduce waste, and enhance informed policy making [5]. A neonatal COS exists for high-income countries (HICs) [6]. Its relevance and applicability to LMICs is uncertain. Differences in disease burden (comorbidities are common and newborns are often on multiple interventions as part of newborn care within the newborn admission ward/newborn unit), resource limitations (e.g. sub-optimal human resources, lack of imaging, follow-up systems), and stakeholder priorities (parents, clinicians, policymakers) may require context-specific outcomes. 

### Isof COS developed in HICs to LMICs possible?

By 2020, only four of 370 global COS originated from LMICs, none from Kenya [7]. Existing neonatal COS from HICs [6] lacked adequate input from LMIC stakeholders, and may not adequately address contextual realities and challenges. This study, therefore, aimed to identify neonatal outcomes reported in sub-Saharan African research, explore the priorities of Kenyan stakeholders—including parents, clinicians, researchers, and policymakers—and build consensus on essential outcomes for a contextualised COS. We hope that the findings of this study will improve the quality of neonatal care by aligning research, clinical practice, and policy with contextually relevant outcomes while offering a model for adapting HIC-developed COS to LMIC settings.

## Methodology

### Study design

This study used a qualitative approach guided by the COMET handbook [5] to develop a COS for neonatal care and research in Kenya. The three sequential phases are as follows: (i) a rapid review comparing outcomes reported in neonatal trials from sub-Saharan Africa (SSA) with those included in an existing HIC COS, to establish a baseline for adaptation,(ii) qualitative stakeholder engagement through focus group discussions (FGDs) and key informant interviews (KIIs) with mothers, healthcare providers, and policymakers to explore and prioritise outcomes relevant to the Kenyan context; and (iii) an in-person consensus workshop using the nominal group technique to finalise the COS. We choose a qualitative approach for its capacity to elicit diverse stakeholder perspectives and enable nuanced prioritisation of outcomes.

### Rapid literature review

A rapid review was conducted to map outcomes reported in neonatal trials from sub-Saharan Africa (SSA) and compare them with those included in the existing HIC COS [6]. Relevant studies were identified through a structured search of electronic databases, including PubMed and Pan African Clinical Trials Registry, using search terms related to neonatal care, clinical trials, and SSA. Studies were included if they reported outcomes from randomised controlled trials or quasi-experimental studies conducted in SSA involving neonates. Outcomes were extracted from eligible studies and mapped against the HIC COS to identify areas of overlap and divergence. This process informed the initial outcome list presented during stakeholder engagement and the consensus meeting, ensuring that context-specific outcomes not represented in the HIC COS were captured from the outset.

### Study setting

Mothers and health care workers from two hospitals with newborn units (NBUs) were purposively selected to represent urban and rural settings. These two settings captured varied socioeconomic profiles, disease burden profiles, and health care system capacities. They participated in face-to-face meetings at their workplaces, and for mothers, the FGD was in a centralised venue near the hospital. The policy-making level participants (Ministry of Health officials and researchers involved in neonatal care and research) were based in Nairobi and participated either at their workplaces or online.

### Participant selection

Participants were grouped into three stakeholder categories: NBU healthcare providers (nurses, paediatricians, and medical officers with varying roles in neonatal care); national-level policymakers (Ministry of Health officials and researchers or professional association representatives with direct or indirect involvement in neonatal policy); and mothers of neonates previously admitted to an NBU. Using purposive sampling, participants were selected based on their professional or lived experience, prioritising diversity of perspective over statistical representativeness [8]. Healthcare providers were selected based on their clinical roles within the NBU. Policymakers were identified through their formal involvement in neonatal care policy or research. Mothers were identified through NBU discharge records and follow-up appointment lists, with the assistance of the NBU manager. This approach was designed to access a rich and varied mix of insights into what outcomes matter in neonatal care and why.

### Data collection

Data were collected between March 2023 and July 2023. Semi-structured KIIs were used for healthcare providers and policymakers, a format selected for its flexibility, depth, and ability to minimise group dynamics that might otherwise constrain openness—particularly between nurses and senior doctors. Interviews were conducted in English, either face-to-face at participants’ workplaces or online via Zoom (Zoom Video Communications, Inc.), and lasted 30–45 min. Discussions began with participants’ daily work roles and how they assessed neonatal progress, before introducing the concept of a COS, its utility, and exploring which outcomes mattered most to participants in their respective contexts.

FGDs were used for mothers to encourage open discussion of shared experiences and facilitate peer dialogue. Sessions were held near the hospital, with transport reimbursement and refreshments provided to reduce barriers to participation. FGDs were conducted in Swahili—Kenya’s national language—with the support of two female research assistants (CW and NO) to foster a culturally comfortable environment. Discussions began with participants sharing updates on their children’s progress, followed by an exploration of which neonatal outcomes were most important to them. Each session lasted approximately 2 h.

After obtaining participants’ written informed consent, all KIIs and FGDs were audio-recorded on an encrypted audio recorder and supplemented by field notes taken during and immediately after sessions.

Topic guides for both KIIs and FGDs were based on a literature review and findings from two prior online surveys that explored the use of COS in LMICs [9],[10]. The guides used open-ended questions and prompts to stimulate conversation and allow unanticipated topics to be discussed. Guides were piloted within the authorship team, reviewed iteratively as data collection progressed, and refined to ensure questions remained responsive to emerging findings. All sessions concluded with participants rating outcomes from the literature and the HIC COS on a 1–10 scale to support triangulation of qualitative findings.

Additional file 1 shows the sample guides (see Additional file 1).

### Reflexivity

JK conducted all KIIs and FGDs. JK is a male with a background in evidence synthesis at KEMRI (2011–2016) and monitoring and evaluation at the Ministry of Health (2016–2021), JK believes that standardised outcomes are essential, not only for guidelines development but also for tracking service delivery. He acknowledged these beliefs at the outset of data collection and actively worked to set them aside throughout, to avoid imposing assumptions on participants or influencing data interpretation. To encourage openness during the interviews, he clarified that he was a PhD student rather than a Ministry of Health official, including this information in participant information sheets and verbal introductions.

He had prior training in qualitative methods (University of Liverpool, KEMRI-Wellcome Trust), which guided the qualitative approach. Reflexivity was maintained throughout by critically examining his own biases, sharing emerging data with co-authors, and debriefing after every few interviews to refine both the questions and the interviewing approach.

### Data management and analysis

FGDs were translated into English, while KIIs (conducted in English) required no translation. Transcription was in the intelligent verbatim format, omitting filler words (e.g. “ums”, “aah”) and repetitions, anonymised, checked for accuracy and stored securely on a password-protected computer. Field notes aided subsequent analysis and contextualisation of transcript content.

Transcripts were uploaded to NVivo 14 Pro software for management and analysis [11]. Analysis was inductive and thematic, informed by Braun and Clarke’s six-phase process framework: familiarisation with the data, generating initial codes, searching for themes, reviewing themes, defining and naming themes and producing the final report [12]. JK developed the initial codebook after reviewing an early transcripts. Codes were then refined iteratively (e.g. “feeding” was reclassified under “weight gain”) as more transcripts were analysed. We analysed the KII data concurrently with data collection to help further refine the interview process and inform our probing questions for the FDGs.

The reporting of the methods, findings and analysis of the qualitative findings is guided by the consolidated criteria for reporting qualitative research (COREQ) [13].

### Consensus process

#### Outcome identification

The initial outcome list was drawn from three sources: (i) the neonatal COS developed for HIC settings [6],(ii) outcomes reported in neonatal studies from SSA [14],[15],[16],and (iii) outcomes identified through KIIs and FGDs. Outcomes were compiled, deduplicated, and grouped thematically prior to the meeting.

#### Setting and participants

The consensus meeting was both study hospitals with transport costs reimbursed. A purposive sample of 25 participants was invited to ensure broad multidisciplinary representation, including mothers of previously admitted neonates, a representative of a Non-Governmental Organisation (a preterm mothers’ network), NBU healthcare workers, neonatal researchers, and policymakers. Some participants had taken part in the earlier KII phase. A full description of invitees is provided in Additional file 2.

#### Consensus meeting process

The meeting focused on refining the existing set of outcomes; no new outcomes were introduced. JK led proceedings with support from DG, opening with presentations on COS and their importance, the importance of patient and public involvement, an overview of the three-source outcome list and the consensus-building process.

#### Data collection and analysis

Although it would have been desirable to conduct anonymous voting on an online platform such as Delphi or Mentimeter [17], we were unable to do so due to time and resource constraints. However, we used a manual pencil-and-paper version of anonymous voting to minimise social desirability bias. Each participant received outcome strips with plain-language descriptions and three response options: “Yes”, “No”, or “Not sure”, with no participant identifiers. Following open plenary discussion of each outcome, papers were collected, tabulated, and converted to percentages. A pre-specified decision rule was applied: outcomes receiving ≥ 70% “Yes” votes were included in the final COS,those below this threshold (“Yes” by < 70%) were excluded [18].

## Results

### Qualitative study

#### Participants

Focus group discussions

Two FGDs were conducted with a total of 15 mothers. FGD 1 (Hospital 1 (rural hospital)) had five participants (eight were invited), and FGD 2 (Hospital 2 (urban hospital)) had ten participants (all invited). Mothers were accompanied by their babies, aged between 2 months and 3 years. Six mothers were accompanied by their babies, who were less than 1 year old. One participant in FGD 2 was accompanied by a helper who did not participate in the discussion but was available to enter the room to assist with the baby. NBU stays ranged from 1 to 8 weeks, with several mothers having experienced Kangaroo Mother Care (KMC) during their baby's admission (Table 1).

**Table 1:** Length of NBU and KMC stay participants in the focus group discussions

Participant	Length of NBU stay
**FGD 1 (Hospital 1 rural hospital)**	
R1	1 week
R2	3 weeks
R3	6 weeks
R4	8 weeks
R5	2 weeks
**FGD 2 (Hospital 2, urban hospital)**	
R1	4 weeks
R2	2 weeks (1 week KMC)
R3	1 week (0.5 weeks KMC)
R4	8 weeks
R5	7 weeks (3 weeks KMC)
R6	4 weeks
R7	4 weeks (2 weeks KMC)
R8	3 weeks (1 week KMC)
R9	4 weeks (1 week KMC)
R10	4 weeks (3 weeks KMC)

*NBU*, newborn unit; *KMC*, Kangaroo Mother Care

#### Key informant interviews

Twenty-two stakeholders were invited, and 17 participated in KIIs. Participants included nurses, medical officers, paediatricians, a neonatologist, and national-level policymakers from the Ministry of Health and research institutions. Experience ranged from less than 1 year to 12 years (see Table 2).

**Table 2:** Stakeholder characteristics of participants in the key informant interviews

Stakeholder number	Stakeholder type	Main role	Experience (years)	Setting	Interview method
R1	Medical officer	Clinical care in the NBU	2	Rural	Face-to-face
R2	Nurse	NBU in charge (administrative) and nursing care	11	Rural	Face-to-face
R3	Policy maker	Head of Child Health—MoH	< 1	Urban	Face-to-face
R4	Policy maker	Researcher and clinical care for neonates in a public facility	12	Urban	Online
R5	Policy maker	Researcher in child health and private clinical practice in neonates	12	Urban	Face-to-face
R6	Policy maker	Health Information System—MoH	10	Urban	Online
R7	Nurse	Nursing services in the NBU	3	Rural	Face-to-face
R8	Nurse	Nursing services in the NBU	9	Rural	Face-to-face
R9	Paediatrician	Clinical care of children, including NBU	< 1	Rural	Face-to-face
R10	Nurse	NBU in charge (administrative) and nursing care	9	Urban	Face-to-face
R11	Neonatologist	Clinical care of neonates in the NBU	7	Urban	Face-to-face
R12	Nurse	Nursing services in the NBU	3	Urban	Face-to-face
R13	Nurse	Nursing services in the NBU	5	Urban	Face-to-face
R14	Nurse	Nursing services in the NBU	2	Urban	Face-to-face
R15	Policy maker	Coordination of newborn care—MoH	5	Urban	Online
R16	Policy maker	Head of Research—MoH	3	Urban	Online
R17	Nurse	Nursing services in the NBU	4	Urban	Face-to-face

*NBU*, newborn unit; *MoH*, Ministry of Health

#### Qualitative findings

FGDs and KIIs were conversational and responsive, and participants raised topics beyond outcome measurement—including health facility organisation, staffing levels, availability of health products, and infrastructure adequacy. The following sections focus on outcomes, as these were the primary study objective, with emphasis on those rated most highly by participants.

#### Neonatal survival/mortality

Survival was rated the most important outcome by all participants, with an average score of 10. Stakeholders across all groups described it as the fundamental objective of any health system, with no meaningful distinctions among stakeholder groups in this regard.

Yes. That one is a number one. That one is important because regardless of the condition you have, you have to survive. (Health researcher, R4)

Nurses emphasised the emotional and community significance of neonatal survival, framing death as not only a personal loss but a generational one:

It is her joy to go home with a healthy baby and a living baby, rather than going home with a dead body. And also, for the community — if we continue losing babies, we will not have people for tomorrow. (Nurse R8, Hospital 1)

Mothers linked survival directly to the quality of NBU care received, expressing gratitude for interventions they believed had saved their babies’ lives:

So, I was saying that if I hadn’t come here that day, I wouldn't have my baby. Honestly, they take good care of the babies. (Mother R2, Hospital 1)

Mortality was identified as most likely among very low birth weight and preterm babies, and those with comorbidities such as sepsis and prematurity, which were described as common presentations in both hospitals.

#### Length of hospital stay

Length of hospital stay was universally rated as critical, with an average score of 9. Nurses highlighted its psychosocial implications for mothers, including risk of job loss, depression, and reduced breast milk production:

The more they stay, all the implications — these mothers have other babies, other things; some are working, and some get even sacked or subjected to unpaid leave. So, it’s very important; it’s a key thing. (NBU in charge, R2, Hospital 1)

Policymakers described length of stay as a useful proxy indicator for quality of care, with prolonged admissions suggesting gaps in neonatal management and increased exposure to hospital-acquired infections:

Length of hospital stay in the newborn is an outcome of management that is very important… it can also indirectly lead to best management in that facility. (Policymaker R16, MoH)

The longer you stay in hospital for us is a measure of maybe something we are missing. (Health researcher, R4)

Mothers described the financial and social burden of extended stays. The national health insurance scheme does not cover mothers’ accommodation costs, and societal expectations created additional pressure to return home quickly:

Friends used to call and ask if I hadn’t left the hospital yet… then they would say, ‘Oh, you’ve been there for so long, we’ll come visit you at home.’ (Mother R6, Hospital 2)

#### Weight gain

This is an important outcome not only for clinicians but also for mothers. Clinicians described it as the primary discharge criterion for low-birth-weight babies—discharge at 1800 g—and as a proxy for feeding adequacy and overall clinical progress.

Clinicians and policymakers indicated that weight is routinely documented in the standard newborn admission record and the comprehensive newborn monitoring chart, completed daily during inpatient stay:

Like for weight gain, for prematurity, for the babies with low birth weight, we always discharge them with 1.8kg. Then we give them a TCA (to come back again/appointment) and a weekly return date. We monitor their weight every week. After 2.5kg, we discharge them to the maternal and child health department. (Nurse R7, Hospital 1).

That one [weighing and documenting] we do it, and so that one is okay. (Policymaker R15, MoH)

Mothers were highly attentive to changes in their babies’ weight and expressed concern when weight loss occurred in the NBU. Some attributed this to the heat of incubators and the frequency of blood draws:

Another thing that makes them lose weight is that they draw a lot of blood; you wonder what all that blood is for. (Mother R6, Hospital 2)

Mothers generally believed that babies gained weight more quickly during KMC than in the incubator, which also influenced their perception of KMC as beneficial beyond infection prevention.

And another thing that makes them lose weight is that they draw a lot of blood, you wonder what all that blood is for. (Mother R6, Hospital 2)

#### Respiratory distress syndrome

Clinicians explained that they were always looking for respiratory distress syndrome (RDS) as a critical clinical indicator of the baby’s born with birth asphyxia and very low birth weight. Clinicians felt that RDS is well documented in the standard newborn admission record form, and the interventions (Continuous Positive Airway Pressure [CPAP] and Mechanical ventilation) provided to the baby are also well documented. Clinicians noted that ideally, as per the guideline recommendation, and resources allowing, all neonates who were below 1500 g should be started on CPAP to prevent RDS, as opposed to waiting for the baby to be in distress and then intervening.

Although constrained, we try to make sure that respiratory distress is resolved, so first of all, you examine the patients and you make sure that there are no issues, and you make sure that things such as oxygen are flowing well and the source of oxygen (Clinician R9, Hospital 1)

Policymakers noted that RDS is often a marker for the quality of intrapartum management as poor labour management may lead to foetal distress and subsequent RDS, linking neonatal outcomes to broader obstetric care quality:

Very key, and very important in tracking it. (Policy maker, R15 MOH)

Mothers felt this was very important, and they could tell when the baby was in breathing distress by seeing the baby placed on oxygen.

Yes, because he was small 1.3kgs he hadn’t been breathing well he was put on oxygen when he was inside there. The oxygen had been removed, but on another occasion I found he had been put back on it until he started breathing independently. (Mother R3, Hospital 1)

#### Sepsis/infection

Although infections were not highly rated as outcomes, clinicians reported that they are a key concern, particularly hospital-acquired infections. Clinicians reported that infection is mostly a product of the length of hospital stay, poor infection prevention and control practices and the limited infrastructure that leads to the sharing of cots and incubators. Mothers from Hospital 2 also echoed concern about the sharing of incubators leading to infections spread.

…… up to five babies would sleep in the same incubator. …..….. There was one who had a cold (flu), and they all got it. (Mother R10, Hospital 2)

, and others, because what we found is that... if one starts developing a fever, you’ll mostly record a fever in the others in the next few days. Yes, so, yes, infections are a major concern. (Medical officer, R1 Hospital 1)

A key diagnostic challenge was the widespread practice of empirical antibiotic treatment for presumed sepsis without laboratory confirmation, inflating reported infection rates and complicating accurate outcome measurement:

…… most of our diagnoses of sepsis are very syndromic ….. most of the babies are technically on treatment for sepsis in the unit. As a cover. (Health researcher, R5)

……….on admission we can do it as a ‘cover-up’ provision of antibiotics so as to protect the child against any infection (Nurse R14, Hospital 2)

Infections were more prevalent in the urban hospital than in the rural hospital, potentially reflecting differences in patient volume, infrastructure, and infection control capacity.

There were further outcomes that are not currently assessed or documented in participants as important to document, as they are part of family-centred care. These were:

#### Pain

Pain was recognised as important by all stakeholder groups but is not routinely assessed or documented in either hospital. Clinicians described informal and inconsistent methods of pain assessment—sternum rubbing, observing grimacing or crying in response to injections—and acknowledged the absence of validated, standardised assessment tools:

It is also important to us, but I don’t think everyone has that capacity or ability to maybe scale the level of pain that this baby will be on or even detect that this baby is in pain. So, it’s important to me, but we need people to have some training on this. (Clinician R9, Hospital 1)

There isn’t a standard approach for it even though there should be, like the pain scales where we can look at the baby's face ………. You can give them a gloved finger to suck on or their own hand so they can calm down. (Clinician R11, Hospital 2)

It’s like you, when the doctor sees that you feel sorry for the baby [when getting the injection], they tell you to leave the room. (Mother R3, Hospital 1)

Policymakers and researchers also believe that pain is an important outcome that is currently not receiving the attention it deserves in neonatal care by clinicians.

Pain, and stimuli, are very important but not very much consideration is being done in our Kenyan situation they [Clinicians] feel it’s not important. (Policymaker R3, MOH)

Mothers expressed distress at witnessing painful procedures and sometimes left the room during blood draws or injections:

It's like when the doctor sees that you feel sorry for the baby when getting the injection, they tell you to leave the room. (Mother R3, Hospital 1)

There is also no standard way of managing the pain in the neonates; some clinicians reported that they would provide paracetamol, others would allow the baby to suck on a gloved finger, while others would ask the mother to breastfeed the baby when a painful procedure is being undertaken.

#### Future well-being and cognitive development

Mothers expressed strong confidence that their children would develop normally, drawing on religious faith and peer support from other mothers whose NBU-admitted babies had grown without complications.

But God is good, He brought something like the NBU, He knew it could help other human beings, so through God and the NBU, we have no worries about our children. They will be fine, they will be healthy, just like their parents. (Mother R1 hospital 1)

From the perspectives of the clinicians and policymakers, though this was a key outcome/indicator, it is one they viewed as very hard to measure, as the current health system does not have a way of following up the children beyond the hospital stay and outpatient clinic visits, which typically would cease after the first 2 years of life. The policymakers, however, indicated that the Kenyan government is rolling out a new digital system that will assign a unique identifier to every neonate and enable longitudinal tracking of developmental milestones, beyond hospital discharge.

#### Cost implication

While care for children under five is nominally free in public facilities, clinicians and mothers reported substantial out-of-pocket costs. Preterm babies with comorbidities frequently require long-term supplementation, specialist follow-up, and laboratory tests that are unavailable at admitting hospitals, necessitating referrals to higher-cost facilities. The NHIF covers only a portion of private-sector costs, and private insurance is rapidly exhausted by prolonged admissions:

Even after being discharged, you are given tests to do, and some of those tests are expensive. Most of the time, you have to go to private facilities, and it’s quite expensive. (Mother R4, Hospital 2)

Mothers also reported purchasing formula milk when breast milk production was insufficient—attributed to stress and inadequate nutrition during hospitalisation—as hospitals do not routinely procure formula feeds.

#### Satisfaction with family-centred care

Mothers felt that hospitals could provide more psychosocial support to them, adequate communication from clinicians during neonates admission, peer-to-peer support amongst mothers of babies admitted to the NBU, and access to adequate food. Experiences differed markedly between hospitals. Mothers in the urban hospital (Hospital 2) described poor communication and feeling mistreated, while mothers in the rural hospital (Hospital 1) praised clinicians for compassionate and explanatory care.

In our facility, our mothers are not satisfied at all. In the morning, you’ll find they give me half a cup of tea and two slices of bread, and even that tea is cold. They give it to you with a lot of ‘noise’ [complaining to her that you are bothering them]. So, these mothers should be taken care of very well, they should be satisfied, get milk, breastfeed, and go home and not quarreled with. (Mother R2, Hospital 2)

…….. the doctor told me that if I ever come and find her extubating, I should hold her like this and put it back. So, whenever I would go and find her extubating, I would put it back. (Mother R5, Hospital 1)

Mothers also felt that KMC equipped them with the skills needed to care for their babies after hospital discharge.

……….. There was counselling, and sometimes a nurse would come and counsel us. People like the mentor mother, who had the experience, would tell us what it would be like to go home and stay with the baby. So, you didn’t even have a hard time with the baby. (Mother R6 Hospital 2)

#### Consensus meeting

##### Participants

Thirteen individuals participated in the consensus meeting: mothers (*n* = 3), paediatricians (*n* = 2), neonatologists (*n* = 2), health records officers (*n* = 2), nurses (*n *= 2), researchers (*n *= 1), and a national-level policymaker (*n *= 1). Two participants (a national-level policymaker and 1 neonatologist) did not complete the voting process due to prior commitments.

##### Outcome discussions and voting

Sixteen outcomes were presented for discussion (see Additional file 3). There was universal consensus (100%) on the inclusion of five outcomes: survival, length of hospital stay, ability to feed/weight gain/growth, cognitive ability later in childhood, and visual impairment/RoP.

Deliberation on these focused on the standardisation of definitions and the grouping of related domains. Feeding ability—encompassing breastfeeding and alternatives such as cup feeding—was agreed to influence weight gain and growth and was therefore grouped within a single outcome domain. RoP was agreed to represent a subset of visual impairment, and the two were merged accordingly, with participants noting that, in the Kenyan context, where preterm births are common and RoP screening capacity is limited, a combined visual impairment domain was more pragmatic.

The other eleven outcomes were discussed individually before voting. Seven outcomes were endorsed for inclusion: impact on mothers and wider family, financial costs to the mother, pain, adverse events due to medicines, RDS, quality of life, and sepsis/infection. Four were excluded: future well-being, jaundice, necrotising enterocolitis (NEC), and the ability to touch/palpate.

Discussions of the included outcomes centred on measurement feasibility as much as on clinical importance. Regarding pain, participants agreed it was important but noted the need for standardised assessment tools before it could be measured reliably. For quality of life, the group acknowledged challenges in long-term follow-up but agreed it should be retained in the COS as an aspirational outcome, with measurement methods to be determined. For sepsis/infections, the group acknowledged diagnostic limitations but agreed that, given the burden, inclusion warranted a pragmatic case definition suited to low-resource settings. For adverse events due to medicines, participants noted the importance of capturing them, particularly given the widespread use of empirical antibiotics. Outcomes excluded from the COS were rejected primarily on grounds of feasibility rather than importance. Future well-being was excluded due to the absence of structured long-term follow-up systems, though participants noted this may change with the planned national digital tracking platform. NEC was excluded because participants felt it could not be reliably diagnosed in lower-level facilities without specialist equipment, risking underreporting. Jaundice was excluded on the basis that it affects only a subset of neonates and is routinely resolved during admission, limiting its utility as a core outcome. The ability to touch/palpate was excluded because participants agreed that cognitive ability, if defined comprehensively, would encompass tactile and neurological assessments.

Stakeholder groups exhibited divergent priorities throughout deliberation. Mothers consistently emphasised non-clinical outcomes—family impact, financial burden, and psychosocial well-being—while clinicians prioritised physiological and measurable outcomes. Researchers and policymakers valued both domains. Steps were taken throughout the meeting to ensure mothers’ voices were heard, including structured opportunities to contribute during each outcome discussion and active facilitation to prevent more senior participants from dominating deliberation.

#### Core outcome set

The final COS comprises twelve outcomes: survival, cognitive ability, visual impairment/RoP, adverse events due to medication, respiratory distress, quality of life, sepsis/infections, length of hospital stay, ability to feed/weight gain/growth, pain, cost of care and impact on mothers and the wider family.

**Fig. 1:**
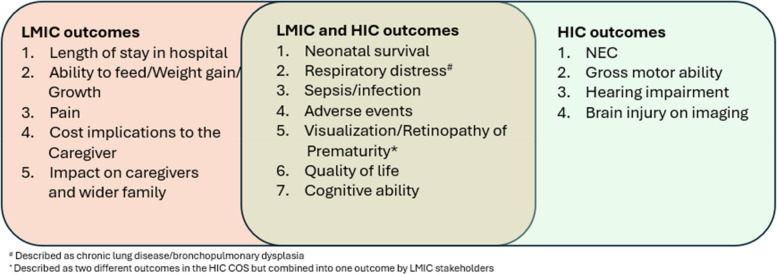
Congruence with HIC COS Webbe et al

A visual inspection indicates substantial overlap between the COS developed for HIC and the outcomes identified for Kenya. This is as shown in Fig. 1.

## Discussion

### Main findings

This study assessed whether a COS developed in an HIC context could be adapted or adopted for use in an LMIC, using Kenya as a case study. Through Rapid review of literature, KIIs, FGDs, and a structured consensus meeting, sixteen outcomes were identified and subjected to a consensus process, yielding a final COS of twelve outcomes: survival, length of hospital stay, ability to feed/weight gain/growth, cognitive ability, visual impairment/RoP, impact on mothers and wider family, financial costs to the mother, pain, adverse events due to medicines, RDS, quality of life, and sepsis/infections.

Seven of the twelve outcomes overlap with the HIC COS [6], confirming that cross-context adaptation of an existing COS is feasible. However, adaptation is a process, not an outcome in itself [5]. The value of an adapted COS depends ultimately on whether it generates more consistent and contextually relevant evidence, a determination that requires continuous evaluation as the COS moves from development to implementation. Definitional modifications were necessary to reflect local diagnostic capacity,for example, RoP was merged into visual impairment rather than retained as a standalone outcome, and RDS was defined more broadly than the HIC COS definition of chronic lung disease or bronchopulmonary dysplasia. These adaptations reflect pragmatic responses to resource constraints rather than evidence of conceptual equivalence: this distinction is key when undertaking cross-context comparability.

All stakeholders universally prioritised neonatal survival, rating it as the most critical outcome. This is unsurprising, as any health system’s primary role is to ensure patients survive. Kenya still faces challenges in reducing neonatal mortality, which remains high (21 per 1000 live births) [3]. Weight gain emerged as the primary indicator of clinical progress, reflecting the high burden of low-birth-weight admissions in Kenyan newborn units [19]. Findings align with those from Nigeria, where parents similarly prioritised breastfeeding, growth, and financial cost [14], suggesting shared LMIC health system priorities across different country contexts [20].

Additionally, part of the adaptation needs to address how to measure, including the selection of time points, especially for non-clinical outcomes. This is especially critical, given that the health systems in most LMICs often lack the capacity to track the neonate beyond the first 2 years of life. Some of the outcomes included in the LMIC COS (pain, growth/ability to feed) were also discussed in the HIC COS, although they were not included in the HIC COS, further showing the similarity of outcomes.

### From development to implementation

It was interesting to note the difference between how clinicians (doctors and nurses) and researchers conceptualise and prioritise outcomes. Clinicians approached outcomes primarily through the lens of immediate bedside management: what is observable, measurable with available tools, and actionable within existing resource constraints. This is appropriate to their role, but it is important to acknowledge that clinicians are not routinely trained to evaluate the broader research implications of outcome selection (including how the choice of outcomes shapes the evidence base, determines what remains unmeasured, and influences future policy [21]. The responsibility for analysing research impact lies primarily with researchers and research-trained policymakers, who are better positioned to assess how outcomes function across studies and contribute to generalisable knowledge.

This distinction was evident during the consensus meeting, where clinicians and mothers tended to prioritise outcomes based on immediate medical or physiological, while researchers and policymakers engaged more explicitly with questions of measurability, standardisation, and research utility. This gap between clinical practice and research evaluation is well documented in implementation science and underscores the need for structured collaboration between practitioners and researchers [22],[23]. This has to be continuously applied in future COS development. In this study, the absence of Ministry of Health policymakers during voting represents a missed opportunity for research-informed policy dialogue. Future exercises should formalise these roles, ensuring researchers lead on outcome definition and measurement standards while clinicians contribute contextual knowledge of feasibility.

### What are the opportunities?

The inclusion of pain as a core outcome is both clinically justified and contextually urgent. Routine procedures often cause pain in neonates, making effective assessment and management critical [24]. Yet evidence from African settings highlights how far practice lags behind intent. Studies from sub-Saharan Africa confirm that neonatal pain is systematically underassessed and undermanaged in clinical practice [25],[26],[27]. Mothers emphasised its significance from an empathetic perspective and the need to console the neonate when painful procedures are being undertaken [28]. Several African studies showing that structured pain assessment tools were used in fewer than a third of neonatal units surveyed, with non-pharmacological strategies (including breastfeeding and non-nutritive sucking) inconsistently applied despite available guidance [26],[27],[29]. Findings from these studies are consistent with this picture: clinicians described informal and variable pain assessment methods, policymakers acknowledged pain was not receiving adequate attention, and no standardised assessment or management protocol existed in either study hospital. The inclusion of pain in the Kenya COS, therefore, signals intent, but meaningful implementation requires investment in validated assessment tools, staff training, and the dissemination of existing locally relevant clinical guidelines for procedural pain management, of which only a few study participants were aware [27].

Incorporating African evidence into future COS work would strengthen the contextual grounding of outcome definitions and measurement approaches.

Additionally, COS adaptation must engage with existing frameworks already embedded in LMIC health systems, like the WHO Essential Newborn Care guidelines; Kenya Basic Paediatric Protocols [30],[31] provide a foundation for standardised practice across resource-limited settings and Kenya, and COS outcome domains should align with these guidelines’ measurement indicators as far as possible to facilitate integration into routine data collection. Similarly, resuscitation protocols such as the Helping Babies Breathe (HBB) programme, which has demonstrated significant reductions in mortality in sub-Saharan African settings [32], and Basic Obstetric Protocols 2026 in Kenya [33] offer existing implementation infrastructure into which COS outcomes related to birth asphyxia and RDS could be embedded.

The preference for broad, non-disease-specific outcomes in this study—compared to the more condition-specific focus of the HIC COS—likely reflects both the high comorbidity burden in Kenyan NBUs [19] and the limited specialist capacity at lower-level facilities and rural health facilities. The exclusion of NEC, brain injury on imaging, hearing impairment, and general gross motor ability illustrates this directly: participants felt it could not be reliably diagnosed without specialist equipment, risking systematic underreporting. Researchers must make this distinction explicit—outcomes may be excluded not because they are unimportant, but because the health system cannot yet measure them—to avoid misrepresenting the scope of the COS.

### What do we need to consider during the adaptation process?

Based on these findings, the following considerations are worth thinking through during the adaptation process.

Patient-to-clinician ratios and workforce planning. Staffing levels in NBUs directly affect both survival outcomes and the quality of outcome monitoring. In both study hospitals, nurses described patient loads that constrained time for structured assessment, including pain evaluation and developmental monitoring. Evidence from LMICs indicates that nurse-to-patient ratios are independently associated with neonatal survival, and research on optimal patient-to-clinician ratios in African newborn units remains needed [34]. Workforce planning should be incorporated into COS implementation strategies, recognising that accurate outcome measurement is labour-intensive and cannot be achieved without adequate staffing.

Phased and context-sensitive implementation. Findings revealed meaningful differences between the urban and rural study hospitals in infection rates, diagnostic capacity, staffing, and mother-clinician interactions. These disparities reflect broader structural inequities in LMIC health systems and indicate that a uniform implementation approach is unlikely to be feasible across facilities with varying capacities [35]. A phased approach would be sensible, beginning with outcomes measurable at all facility levels (such as survival, weight gain, and length of hospital stay) before introducing outcomes requiring specialist assessment in higher-capacity settings. This phased approach is consistent with COMET Initiative guidance on COS implementation in low-resource settings and has been shown to improve quality of care using the Clinical Information Network in Kenya [5],[36].

Significant gaps in knowledge and practice were identified across several outcome domains, most notably pain assessment and management. Sustainable improvement in outcome monitoring requires investment in structured, recurring on-the-job training rather than episodic workshops. Research is needed on the frequency, scale, and perceived effectiveness of on-the-job training in Kenyan NBUs, as well as on the modalities most effective for building research capacity among frontline clinicians. Training should explicitly distinguish between clinical/medical outcome monitoring and research outcome (impact) measurement, equipping clinicians to contribute meaningfully to research conducted in their facilities.

### Strengths and limitations

This study is among the first to undertake systematic COS adaptation for neonatal care in sub-Saharan Africa. Its multistakeholder design (incorporating mothers, clinicians, researchers, and policymakers) strengthens the contextual legitimacy of the final outcome set, and the combination of qualitative methods with a structured consensus process enables nuanced prioritisation that a survey-only approach would not capture.

Several limitations should be noted. First, only mothers participated in FGDs, meaning fathers' perspectives are absent. Secondly, demographic and socioeconomic data from mothers were not collected in depth, limiting the interpretation of how socioeconomic factors shaped their priorities and potentially obscuring some of their perspectives and the adequacy of the sampling. Further exploration of demographic data would be valuable if similar work were to be undertaken. Thirdly, online KIIs may have limited the ability to capture non-verbal cues. Fourthly, during the consensus process, all stakeholder groups were in the same room, which may have intimidated mothers and limited their contributions alongside healthcare providers and policymakers. We tried to make them feel comfortable by giving mothers opportunities to contribute during discussions and asked other participants to listen to what they had to say. We did this for every outcome. Additionally, one of the mothers was a leader of a network of mothers of premature babies who was used to taking part and contributing in various fora to advocate for preterm babies. Finally, the study was conducted in two Kenyan hospitals, and generalisability to other LMIC settings should not be assumed without further validation.

## Conclusion

This study demonstrates that adapting an HIC neonatal COS for use in an LMIC context is feasible, yielding a twelve-outcome set that reflects both shared clinical priorities and the structural realities of the Kenyan health system. The seven outcomes shared with the HIC COS provide a basis for cross-context comparability, while the five Kenya-specific outcomes capture dimensions of neonatal care absent from HIC-derived frameworks. COS utility requires more than definitional adaptation: it demands investment in workforce capacity, phased implementation sensitive to urban–rural differences, and stronger collaboration between clinicians and researchers to ensure that outcome measurement serves both care improvement and evidence generation.

## Supplementary Information

Additional file 1: Topic guides for the data collection.

Additional file 2: Rationale for the inclusion of key stakeholders in the invitation to the consensus process.

Additional file 3: Outcomes identified from the literature, qualitative interviews, and plain language description.

## Data Availability

The data supporting the conclusions of this study are included within the article and its additional files.

## Abbreviations

COS: Core outcome set
FGD: Focused group discussions
HIC: High-income countries
KEMRI: Kenya Medical Research Institute
KII: Key informant interviews
KMC: Kangaroo Mother Care
LMIC: Low- and middle-income countries
MoH: Ministry of Health
NACOSTI: National Commission for Science, Technology, and Innovation
NBU: Newborn unit
NEC: Necrotising enterocolitis
RoP: Retinopathy of prematurity
