# Supplementary material for: To adopt or adapt an existing COS for neonatal research: qualitative study
Source: Trials. 2026 Jun 8;27:522. doi: 10.1186/s13063-026-09834-w (PMC13466529; doi:10.1186/s13063-026-09834-w)
Supplement: Supplementary file 1 [file 13063_2026_9834_MOESM1_ESM.pdf]

Additional file 2: Rationale for inclusion of key stakeholders in the invitation for the consensus process

S.No	Role	Institution	Part of KIIs	Rationale for inclusion
**Policy makers**				
1	Head of Division of Child Health	Ministry of Health	Yes	For any policy change related to child health, such as an update to admission records, the office endorsement is required.
2	Head of Monitoring and Evaluation	Ministry of Health	Yes	The office tasked with tracking key indicators in the health sector, the agreed outcomes are to be linked to key indicators that need to be tracked.
3	Head of Division of Research	Ministry of Health	Yes	The office that coordinates and provides policy guidance in research within the ministry, if the COS is to be used in research, then it's helpful to have the office endorse the content and process of selection.
4	Paediatrician/Clinical guidelines development committee	Ministry of Health	No	Former head of child health in the Ministry of Health, with vast experience in the development of paediatric clinical guidelines using the GRADE system, hence quite familiar with the need for prioritization of outcomes
5	Health information system specialist	Ministry of Health	Yes	Officer overseeing the Kenya Health Information System (National Routine reporting system) in case there is need for change in some of the data collection tools and registers then this office would be crucial as the driver of this process
6	Community Health specialist/Monitoring and evaluation	Ministry of Health	No	Community health work is key especially for the documentation and reporting of the follow-up of some of the outcomes and possibility of provision of information about the long-term nonclinical outcomes.
7	Newborn Program Manager	Ministry of Health	Yes	Newborn programme manager oversees the implementation of newborn care including initiating the development of various guidelines and policy in the newborn space
**Clinicians/Hospital based staff**				
8	Paediatrician	Hospital YYY and XXX	Yes	Clinician based in the hospital providing care to neonates on a day-to-day basis and ensuring there is proper documentation
9	Nursing officer/NBU In charge	Hospital XXX and YYY	Yes	Nursing officer who is the administrative head of the NBU but also providing care to neonates on a day-to-day basis and ensuring there is proper documentation

S.No	Role	Institution	Part of KIIs	Rationale for inclusion
10	Health Records and Information Officer	Hospital XXX and YYY	No	Officers tasked with aggregation of hospital data and submitting it monthly to the Kenya Health Information System. They aggregate data from the NBU data collection tools. They will be useful in helping to implement any changes for outcome data that may be collected.
**Universities/Research**				
14	Neonatologist/Panel of experts for newborn care	University ZZZ	No	Within the Child Health Technical Working group in the Ministry, there is a sub-group (Panel of experts in newborn care) this panel provides technical inputs in policy and guidelines changes in the newborn area. Additionally, he is well-versed in the GRADE system of clinical guidelines development, having been involved in the guidelines development process. He also chairs the research arm of the Kenya Paediatric Association.
15	Paediatrician/Researcher in the Clinical Information Network	University ZZZ/Research Institution	Yes	A senior lecturer, conversant with the GRADE system of clinical guidelines development having being part of the guidelines development process. Additionally, he is part of the Clinical Information Network that is being implemented across several hospitals and is collecting data on newborn outcomes for the last couple of years. Involved in newborn research work and practices as a clinician in the ZZZ University training hospital as part of his University Teaching
16	Paediatrician/Researcher in the Clinical Information Network	Research Institution	Yes	A researcher who is conversant with the GRADE system of clinical guidelines development, having been part of the guidelines development process. Additionally, he is part of the Clinical Information Network that is being implemented across several hospitals and is collecting data on newborn outcomes for the last couple of years. He also undertakes private clinical practice
17	Paediatrician	University WWW	Yes	A senior lecturer, well conversant with the GRADE system of clinical guidelines development, having been part of the guidelines development process. He practices at Hospital YYY (Link training facility for the University) and teaches students.
18	Researcher	Knowledge Management	No	Researcher working on knowledge management in KEMRI and Cochrane Kenya. This will be one of the potential users of the COS that is agreed upon.
**Professional associations and Regulators**				
19	Paediatrician	Kenya Paediatric Association	No	Buy in of professional associations would be key. As previously suggested, this will be one of the avenues for disseminating not only the consensus work outputs but also the entire PhD output work. This is a national association of paediatricians.

S.No	Role	Institution	Part of KIIs	Rationale for inclusion
20	Nursing officer/Newborn programme implementation manager	Neonatal Nursing Association of Kenya	No	Buy in of professional associations would be key. As previously suggested, this will be one of the avenues for disseminating not only the consensus work outputs but also the entire PhD output work. This is a national association of nurses working in the neonatal space. The officer is also the programme manager of a programme that is supporting a few hospitals within the Clinical Information network (Hospitals YYY and ZZZ included). Some of the support consists of provision of phototherapy machines, incubators etc.
21	Clinical Trial Unit	Pharmacy and Poisons Board^	No	This board is the regulator of all clinical trials in Kenya. If the COS is to be entrenched in the regulatory framework, then we need to have an officer from the board.
**Caregivers/Parents**				
22	Mother	A non-profit making organization*	No	To get a perspective of mothers who have had preterm babies
23	Mother	Hospital YYY	No	To get a perspective of mothers who have had preterm babies
24	Mentor mother	Hospital YYY	No	To get a perspective of mothers who have had preterm babies

^(regulatory body for all clinical trials), *(A Non-Profit Making Organization) for mothers who have had preterm babies
