# Supplementary material for: To adopt or adapt an existing COS for neonatal research: qualitative study
Source: Trials. 2026 Jun 8;27:522. doi: 10.1186/s13063-026-09834-w (PMC13466529; doi:10.1186/s13063-026-09834-w)
Supplement: Supplementary file 2 [file 13063_2026_9834_MOESM2_ESM.pdf]

Additional file 3 - Outcomes identified from the literature, qualitative interviews, and plain language description

No	Proposed outcomes for neonatal COS LMICs	Definitions	Plain language definition
1	Survival/Mortality	Overall survival	The time until someone dies from any cause
		Survival	How long do people live after leaving the ICU?
		Mortality	The event of death within a particular time frame.
		All-cause mortality	Death by any cause
		Death from any cause	This outcome refers to the death of someone from any cause, including prostate cancer
		Maternal mortality (death)	Maternal mortality is the death of a woman while pregnant or within 42 days of the end of pregnancy.
		Maternal death	Death of the mother
		Neonatal death	A neonatal death is defined as a death during the first 28 days of life
		Livebirth	The birth of a live baby
		Miscarriage	The spontaneous loss of a pregnancy before the fetus can survive outside the uterus; most common in the first trimester
		Stillbirth	A birth in which the baby is born dead after 22 weeks gestations or with a birth weight of 500 g. This definition may vary slightly across various jurisdictions
		Intrapartum death	The death of a baby during labour and birth
		Mortality excluding suicide	Death from causes other than suicide
		Suicide	Death due to suicide
2	Cognitive ability	Cognitive function	Ability to think clearly including concentration, understanding and remembering things

		Cognitive function/symptoms	Do you have problems with memory, communication, concentrating, or understanding instructions?
		Cognition	Impact on concentration (e.g. at work or at school, or in leisure activities)
		Cognitive impairment	This relates to whether a baby or child is able to think and learn like other children their age.
3	Visual impairment/Retinopathy of prematurity	Processing visual information	Being able to recognise, understand and interpret objects is…
		Retinopathy of prematurity	This refers to abnormal development of the blood vessels in the eyes which can lead to impaired vision or blindness and may require invasive treatment
4	Pulmonary function/Coming off oxygen/Normal breathing	Pulmonary function/symptoms	Do you have trouble breathing, shortness of breath, or cough?
5	Sepsis	Sepsis/septic shock	The event of developing severe infection in the blood resulting in fever, increased heart rate with a fall in blood pressure or other organ dysfunction.
		Neonatal sepsis	Sepsis is a severe infection in the bloodstream that can cause damage to organs or death. It can be divided into early onset and late onset sepsis. Early-onset neonatal sepsis (EOS) occurs at ≤72 h in infants hospitalized in the neonatal intensive care unit (NICU) and <7 days in term infants. Late-onset sepsis (LOS) is sepsis occurring after 72 h in NICU infants and 7 days of life in term infants.
6	Necrotizing enterocolitis	Necrotising enterocolitis	Necrotising enterocolitis (NEC) is an uncommon serious disease affecting the gut of babies. It is more common in premature babies. It can cause life-long disability and death. Surgery can be required to treat it.
7	Ability to feed/Breast feed	Exclusive breast-feeding	This refers to babies who have only ever received breast milk as milk feeds

		Establishment of breastfeeding	When the mother's condition would not get in the way of her breastfeeding her baby
8	Hearing impartment	Vision & hearing	Being able to see, hear and understand is…
9	Adverse events due to medicines	Adverse reaction	Any bad or unexpected thing that happens during the time a treatment is being tested and are thought to be a result of that treatment being tested
		Serious adverse effects of intervention	When the treatment given causes unintended effects on the woman's body that lead to serious harm
		Side effects of intervention	When the treatment given causes effects on the woman's body that are not intended
		Side-effects	The number, severity, impact of side-effects, including physical health, psychological and cognitive effects, including those which reduce sensitivity and make people feel blunted
		Adverse effects of surgical treatments	All types of side effects from surgical treatments (e.g. bleeding, infection, contractures)
		Adverse effects of medical treatments	All types of side effects from medical treatments
		Neonatal side effect of intervention	When treatment given to the mother causes harm or unintended effects on the newborn
10	Jaundice/need for phototherapy	Hyperbilirubinemia requiring intervention	A neonatal complication associated with newborn jaundice when high levels of bilirubin in the blood make a baby's skin look yellow. This can be treated using blue light or blood transfusions (an "exchange transfusion").
11	Pain	Neck pain	Unpleasant physical sensation in the neck
12	Weight gain/Growth		Ability to gain weight or increase in length
13	Length of stay in hospital	Length of stay	The number of days spent in hospital.

		Duration of hospital stay	This outcome is only relevant to an individual who has a procedure requiring them to stay in hospital. It refers to the length of time spent in hospital during and after the treatment.
		Duration of hospital stay	Time child has had to stay in hospital
		Number of hospital bed use days	The total number of days a person spends in hospital over a given period of time
		Length of hospitalisation	How long the mother has to stay in hospital
		Length of hospital stay	This refers to the number of days the mother is in hospital from admission to discharge
		Length of hospital stay	This refers to the number of days the baby is in hospital from birth to discharge
14	Quality of life	Health-related quality of life	A state of health and well-being, which includes physical, mental, emotional and social well-being.
		Quality of life	Child's ability to enjoy normal activities
		Overall quality of life	Quality of life in general related to physical and emotional wellbeing.
		Quality of life	A state of health, happiness, comfort and well-being
		Health related quality of life	An overall measure how a person's health effects their general wellbeing
15	Financial costs.	Feeling financially secure	Feeling financially secure and comfortable with financial arrangements is…
		Financial impact on patient	Are you dealing with hospital bills or lost income from time off work? Do you have to pay someone to help with your care or transportation?
16	Emotional impact on caregiver/wider family	Impact on family and/or caregivers	Do you feel you are a burden on family or friends? How does being sick affect other people in your life?
		Quality of life impact on immediate family	Impact of treatment on immediate family members in terms of their quality of life.

		Impact on family and/or caregivers	Do you feel you are a burden on family or friends? How does being sick affect other people in your life?
		Reaction of family/friends to diagnosis	Knowing that your family and friends accept and understand you with your diagnosis is…
		Carer reaction	The reaction of your carer or care partner to the things you do or say as a result of dementia is… (for example, your care partner could be a family member or another significant person or friend. Things might include forgetfulness or behaving differently than you used to)
