# Supplementary material for: To adopt or adapt an existing COS for neonatal research: qualitative study
Source: Trials. 2026 Jun 8;27:522. doi: 10.1186/s13063-026-09834-w (PMC13466529; doi:10.1186/s13063-026-09834-w)
Supplement: Supplementary file 3 [file 13063_2026_9834_MOESM3_ESM.pdf]

Additional File 1: Topic guides for the data collection

i. Key informant interview guide for health care providers in the hospitals

Tell me about day-to-day activities in the neonatal unit.

For how long have you been working in the neonatal unit?

When a newborn is admitted here what happens? (probe for the admission process including communication to the caregiver/parent

Can you describe the work environment? (probe for availability supplies and commodities; data capture tools etc?

Provide definition of an outcome using an example in plain language (guided by COMET initiative plain language summary).

What would you say are the most important outcomes for a neonate admitted in the NBU? (Probe why they think each is important)

Do you collect the data on the outcomes mentioned above routinely? (probe for measurement of the outcomes)

Describe outcomes from the HIC neonatal COS and ask them to rate or rank them in order of importance (1 [least important] to 10 [most important])

Provide brief description of a COS

What are your perceptions about having a COS for use in your area of work (Probe for perceptions on research and on routine work)

Exit Question

Is there anything else you would like to say in relation to outcomes in neonatal care?

ii. Key informant interview guides for national policy makers

Tell me about your day-to-day activities (probe for activities linked to neonatal health)

What are the most important outcomes that have been prioritized neonatal health? (probe for the process of generation of the outcomes and their implementation, measurement and evaluation)

How often is the data from the routine system and or research inform development of policies (probe for process of data use; how it has worked; what can be changed)

Describe outcomes from the HIC neonatal COS and ask them to rank them in order of importance ((1 [least important] to 10 [most important])

Provide brief description of a COS

A COS is an agreed minimum set of outcomes to be measured and reported in all trials in a specific area like newborns admitted to the hospital.

What are your reactions and thoughts about establishing a COS for neonatal care?

What are the potential benefits/ (or drawbacks)?

Can you suggest key criteria an outcome must fulfil to be included as a "core outcome" – why? (Probe for feasibility of its measurement, clinically relevant, scope)

What do you think needs to be considered in implementing the COS?

What impacts (e.g. research, practice, policy, patient outcomes) do you think the implementation of a COS will have?

What would it take to include an outcome in the routine data collection systems (probe for frequency of review of data collection indicators, process of change, cost implication)?

Exit Question

Is there anything else you would like to say in relation to outcomes in neonatal care?

iii. Mothers FGD guide (English version)

Engagement questions

When did your baby get admitted to the unit?

What has happened since the child got admitted? (Probe for parent and child)

Explorative questions

How do you judge how well your baby is doing?

How do you think the doctors and nurses judge how well your baby is doing?

What makes you concerned about your baby's health? (Probe for things they've been worried about)

What are your expectations/concerns about your baby when you go back home and as they grow?

Provide definition of an outcome using an example in plain language (guided by COMET initiative plain language summary).

What would you say are the most important outcomes for your baby to consider effective care/treatment?

Describe the outcomes from the HIC neonatal care set and ask them to rank the outcomes in order of their importance to them. (1 [least important] to 10 [most important])

Exit Question

Is there anything else you would like to say in relation to outcomes of your baby?
